# Associations Between C-Reactive Protein, Insulin Sensitivity, and Resting Metabolic Rate in Adults: A Mediator Analysis

**DOI:** 10.3389/fendo.2018.00556

**Published:** 2018-09-20

**Authors:** Theresa Drabsch, Christina Holzapfel, Lynne Stecher, Julia Petzold, Thomas Skurk, Hans Hauner

**Affiliations:** ^1^Institute for Nutritional Medicine, University Hospital Klinikum Rechts der Isar, Technical University of Munich, Munich, Germany; ^2^ZIEL Institute for Food and Health, Technical University of Munich, Munich, Germany; ^3^Else Kroener-Fresenius-Center for Nutritional Medicine, Chair for Nutritional Medicine, Technical University of Munich, Munich, Germany

**Keywords:** resting metabolic rate, energy expenditure, C-reactive protein, insulin sensitivity, inflammation, homeostasis model assessment for insulin resistance

## Abstract

**Objective:** Long-term positive energy balance promotes the development of obesity, a main risk factor for type 2 diabetes mellitus (T2DM). While an association between increased resting metabolic rate (RMR) and insulin sensitivity (IS) was shown previously, the underlying mechanisms remain unclear. Aim of the mediator analysis was to investigate the role of inflammation within the association between RMR and IS.

**Methods:** Anthropometric, clinical, and lifestyle data were collected according to standard operating procedures. RMR was measured using indirect calorimetry. Homeostasis model assessment for insulin resistance (HOMA-IR) was used as an IS parameter and C-reactive protein (CRP) was measured to represent the inflammatory status. Statistical analyses were performed using SPSS.

**Results:** The analysis included 782 adults (517 females) with a mean age of 32.4 ± 12.0 years and a mean body mass index (BMI) of 24.6 ± 5.2 kg/m^2^. Regression analysis indicated a significant evidence for associations between RMR and HOMA-IR (ß = 39.3 ± 7.3 kcal/d; *p* ≤ 0.001) and CRP and HOMA-IR (ß = 0.5 ± 0.1; *p* ≤ 0.001) after adjustment for fat-free mass, sex, age, and study site. Results of the mediator analysis did not support the hypothesis that CRP is a mediator for the association between RMR and HOMA-IR. These results did not change after participant stratification according to sex or BMI.

**Conclusion:** A significant evidence for an association between RMR and IS was shown in a large cohort. However, the inflammatory status, determined via CRP levels, was not a mediator within this association.

## Introduction

The Global Burden of Disease consortium estimates that the prevalence of obesity and type 2 diabetes mellitus (T2DM) has more than doubled globally in recent decades (1). Data from the World Health Organization indicates that the global prevalence of T2DM among adults has risen from 4.7% in 1980 to 8.5% in 2014 (2). A positive energy balance, triggered by modern and sedentary lifestyle with low physical activity and high consumption of energy-dense foods is associated with the development of overweight and obesity, which are the two main risk factors for T2DM (3).

Both obesity and T2DM are characterized by not only impaired insulin sensitivity (IS) but also low-grade inflammation (4, 5). Subacute chronic inflammation might promote metabolic disturbances, providing evidence for the designation of T2DM as a chronic inflammatory disease (6, 7). Studies suggest a negative association between resting metabolic rate (RMR) and IS (8–11). In two studies, subjects with obesity and impaired glucose tolerance exhibited higher RMR levels than those with obesity and normal glucose tolerance (10, 12). Furthermore, a significantly positive association between RMR and C-reactive protein (CRP), an acute-phase protein as marker of inflammation, was reported in participants suffering from severe diseases (13, 14). This effect could be explained by the energy costs due to the inflammatory status (15), which is common in persons with T2DM (5, 16).

To date, the role of the inflammatory status within the association between RMR and IS as well as the impact of inflammation on RMR remains unclear. Therefore, the present analysis was conducted to evaluate the role of the inflammatory status, represented by CRP levels, within the association between RMR and IS. In addition, sex- and body mass index (BMI)-specific subanalyses were performed to study potential confounders.

## Subjects and methods

### Subjects

Recruitment started in October 2013 and took place at two equally equipped study sites of the Else Kroener-Fresenius Center for Nutritional Medicine in Munich and Freising-Weihenstephan, Germany. Volunteers were screened for eligibility and included if age was ≥18 years and BMI was ≥18.5 kg/m^2^. Subjects who appeared healthy and showed no history of severe diseases or surgery within the last 3 months and did not have acute physical impairment were eligible. Pregnant and breast-feeding women were excluded. Participants were studied after an overnight fast of about 10–12 h and had to refrain from smoking and physical activity for 12 and 24 h before RMR measurement, respectively. All study procedures were in agreement with the ethical guidelines and were approved by the ethical committee of the Technical University of Munich, Germany (Number 2719/10 S). Written informed consent was obtained from all persons before participation in the study. For the present analysis, participants with overt diabetes, based on self-reporting or with a fasting plasma-equivalent glucose value above 125 mg/dl, were excluded. Furthermore, individuals on medications affecting the sympathetic nervous system such as beta blockers were excluded. For subanalyses, individuals with hypertension (systolic/diastolic blood pressure >140/90 mmHg), defined according to the guidelines of the European Society of Cardiology (17), with dyslipidaemia, with drug intake such as statins or aspirin or with impaired fasting plasma-equivalent glucose levels (IFG) of 100–125 mg/dl, defined according to the German Diabetes Association (18), were excluded.

### Anthropometry, medical history, and lifestyle

Height was measured without shoes in a standing position using a stadiometer (Seca, Hamburg, Germany) and reported to the nearest 0.1 cm. Body composition was assessed by bioimpedance analysis using Tanita BC 418 MA (Tokyo, Japan). Measurements were performed in light clothing and with voided bladder, whereby 1.0 kg was subtracted automatically for clothes. BMI was calculated as the quotient of weight and height squared (kg/m^2^). Using a validated electronic device (Beurer BM70, Ulm, Germany) blood pressure was measured as recommended by the American Heart Association (19) and heart rate was obtained according standard operating procedures. Information on the health status, medication, smoking, and physical activity were assessed using a standardized questionnaire. A four-level graded scale was used for the assessment of physical activity during winter and summer season (0, <1, 1-2, and >2 h/week) (20).

### Clinical parameters

Fasting blood samples were obtained after RMR measurement. Fasting blood glucose level was analyzed from whole blood using a HemoCue® Glucose 201^+^ System with plasma conversion (HemoCue AB, Ängelholm, Sweden). Serum collection tubes were stored half an hour at room temperature before centrifugation at 3,000 rpm with 4°C for further 10 min. Serum samples were analyzed for hs-CRP level by turbidimetry (ADVIA 2400, Siemens Healthcare GmbH, Erlangen, Germany) and insulin level by a chemiluminescence immunoassay (Immulite 2000, Siemens Healthcare GmbH, Erlangen, Germany) by SYNLAB Holding Deutschland GmbH (Augsburg, Germany). Homeostasis model assessment for insulin resistance (HOMA-IR) was calculated as follows:

$$\text{HOMA}-\text{IR}=\;\frac{\text{glucose }\left(\frac{\text{mg}}{\text{dl}}\right)\times \text{insulin }\left(\frac{\mu \text{U}}{ml}\right)}{405}$$

(21).

### RMR measurement

Indirect calorimetry was performed to measure RMR with a breath-by-breath system through a ventilated canopy hood (Quark RMR, Suite Version 10.0e, Cosmed s.r.l., Rome, Italy). All RMR measurements were performed according to a standard operating procedure. The initial 5 min of the measurement were considered as an acclimatization phase before the actual 30 min measurement. The flow rate was assessed for each participant and adjusted to meet a fraction of expired carbon dioxide of 1.0%, as recommended by the manufacturer. The Quark software recorded oxygen consumption (VO_2_) and carbon dioxide production (VCO_2_) resulting from substrate oxidation continuously every 5 min under a ventilated hood with the participant fully awake and motionless. RMR was calculated by the following equation (22):

$$\text{RMR}\frac{kcal}{d}=\;\left[\left(3.941\times VO_{2}\frac{ml}{min}\right)+\left(1.106\times VCO_{2}\frac{ml}{min}\right)\right]\times 1.44\;.$$

The RMR values were recalculated for measurements with VO_2_ and/or VCO_2_ variances equal to or >10.0% by choosing a 4-min window for correction. The results obtained using a 4-min time window are not statistically different to that measured over a 30-min period (23). RMR measurements in which correction was not feasible were excluded from the analysis.

### Data analysis

Characteristics of study participants, and their clinical parameters, were summarized using means ± standard deviation (SD) and percentages (%). The continuous variables were compared between males and females or between individuals with BMI >24.9 kg/m^2^ and normal-weight subjects using Student's paired *t*-tests or Mann-Whitney U-test, as deemed appropriate. Linear regression analyses were fitted to assess the effect of fat-free mass (FFM), BMI, insulin, or plasma-equivalent glucose on RMR. These models were further adjusted for age, sex, study site, and FFM. The regression models were also performed in subgroups according to sex. HOMA-IR and CRP values were not normally distributed and were therefore log-transformed for the regression analysis. A mediator analysis, according to Baron and Kenny (24), examined the mediator role of CRP within the association between RMR and IS in three stages using linear regression models. In the first two stages, the association between RMR and HOMA-IR (independent variable) and the association between CRP and HOMA-IR (independent variable) were assessed. For the mediator analysis to be meaningful, the independent variables should be statistically significant in these two stages. In the final stage, HOMA-IR and CRP were included in the same regression model for RMR. If CRP was acting as a mediator then CRP would be significant and the effect of HOMA-IR would be significantly reduced. Further, the mediator analysis was performed unadjusted, adjusted for FFM, age, sex, and study site and additionally for smoking and physical activity at each stage. In subanalyses (not adjusted for smoking and physical activity) the mediator analysis was performed in subgroups according to BMI categories or after exclusion of individuals suffering from hypertension, or persons with dyslipidaemia, with drug intake such as statins and aspirin, or with IFG levels. Additional analyses were conducted using linear regression models to investigate the interaction of BMI or sex with HOMA-IR as well as that of CRP on RMR. *P*-values ≤ 0.05 were considered statistically significant. All statistical analyses were performed by using SPSS statistical software (SPSS version 23.0, SPSS Inc., Chicago, IL, USA).

## Results

Baseline characteristics of the study participants (*n* = 782, 66.1% females) are shown in Table 1. Mean age was 32.4 ± 12.0 years, mean BMI was 24.6 ± 5.2 kg/m^2^, and mean FFM was 54.5 ± 11.9 kg. Mean systolic and diastolic blood pressure values were 113.8 ± 13.3 and 70.4 ± 8.9 mmHg, respectively. Approximately 4% of the participants (*n* = 35) had an increased blood pressure (>140/90 mmHg). Most participants (77.0%) were non-smokers. Mean RMR was significantly higher in males compared to females (1,803.4 ± 241.3 vs. 1,366.2 ± 171.5 kcal/d, *p* ≤ 0.001). There were no significant differences in age or BMI between males and females (*p* > 0.05). Compared to females, males had a significantly higher FFM (68.9 vs. 47.1 kg, *p* ≤ 0.001) but a significantly lower mean fat mass (13.9 vs. 21.5 kg, *p* ≤ 0.001). Mean HOMA-IR was 1.1 ± 0.9 (range, 0.2–8.6), whereas mean CRP was 0.20 ± 0.35 mg/dl (range, 0.02–5.66 mg/dl) (Table 2). No sex-specific differences in HOMA-IR or insulin levels were observed; however, significantly higher CRP values were found in females compared to males (0.23 vs. 0.14 mg/dl, *p* ≤ 0.001). Mean HOMA-IR and CRP values were significantly higher in individuals with BMI >24.9 kg/m^2^ compared to normal-weight subjects (data not shown).

**Table 1:** Characteristics of study participants.

**Parameter**	**All (*****n*** = **782)**		**Males (*****n*** = **265)**		**Females (*****n*** = **517)**	
	***n***	**Mean ± SD**	***n***	**Mean ± SD**	***n***	**Mean ± SD**
Age (years)	782	32.4 ± 12.0	265	31.7 ± 11.8	517	32.7 ± 12.2
18–24	258	33.0%	91	34.3%	167	32.3%
25–34	280	35.8%	99	37.4%	181	35.0%
35–50	150	19.2%	50	18.9%	100	19.3%
51–64	83	10.6%	20	7.5%	63	12.2%
≥65	11	1.4%	5	1.9%	6	1.2%
Weight (kg)	781	73.4 ± 16.5	265	82.8 ± 14.5	516	68.6 ± 15.3
Height (cm)	781	172.6 ± 9.7	265	182.2 ± 7.1	516	167.7 ± 6.7
BMI (kg/m^2^)	781	24.6 ± 5.2	265	24.9 ± 4.3	516	24.4 ± 5.6
18.5–24.9	511	65.4%	156	58.9%	355	68.8%
25.0–29.9	182	23.3%	86	32.5%	96	18.6%
≥30.0	88	11.3%	23	8.7%	65	12.6%
FFM (kg)	781	54.5 ± 11.9	265	68.9 ± 7.5	516	47.1 ± 4.9
FM (%)	781	25.1 ± 10.3	265	15.8 ± 7.5	516	29.8 ± 8.2
FM (kg)	781	18.9 ± 11.4	265	13.9 ± 9.4	516	21.5 ± 11.5
Systolic BP (mmHg)	779	113.8 ± 13.3	265	120.5 ± 11.3	514	110.3 ± 13.0
Diastolic BP (mmHg)	779	70.4 ± 8.9	265	72.5 ± 9.5	514	69.3 ± 8.5
Heart rate (bpm)	776	60.3 ± 9.3	264	59.6 ± 9.2	512	60.7 ± 9.4
Smoker	72	9.2%	26	9.9%	46	8.9%
Non-smoker	600	77.0%	203	77.2%	397	76.9%
Ex-smoker	107	13.7%	34	12.9%	73	14.1%
RMR (kcal/d)	782	1,514.4 ± 286.4	265	1,803.4 ± 241.3	517	1,366.2 ± 171.5
VO_2_ (ml/min)	782	220.2 ± 43.2	265	262.6 ± 37.6	517	198.5 ± 26.7
VCO_2_ (ml/min)	782	177.2 ± 32.2	265	210.0 ± 26.9	517	160.3 ± 19.1

*Mean ± SD is shown, if not otherwise indicated; n, number; BMI, body mass index; FFM, fat-free mass; FM, fat mass; BP, blood pressure; RMR, resting metabolic rate; VO_2_, volume oxygen; VCO_2_, volume carbon dioxide*.

**Table 2:** Clinical parameters.

**Parameter**	**All (*n* = 782)**	**Males (*n* = 265)**	**Females (*n* = 517)**
	**Mean ± SD**	**Mean ± SD**	**Mean ± SD**
CRP (mg/dl)	0.20 ± 0.35	0.14 ± 0.23	0.23 ± 0.39
Fasting insulin (μU/ml)	5.0 ± 4.0	4.9 ± 3.9	5.0 ± 4.1
Fasting blood glucose (mg/dl)	87.9 ± 9.6	89.1 ± 9.2	87.3 ± 9.7
HOMA-IR	1.1 ± 0.9	1.1 ± 0.9	1.1 ± 1.0

*Mean ± SD is shown, if not otherwise indicated; n, number; CRP, C-reactive protein; HOMA-IR, homeostasis model assessment insulin resistance*.

### Associations between RMR and BMI, FFM, insulin, or plasma-equivalent glucose

**Figure 1:**
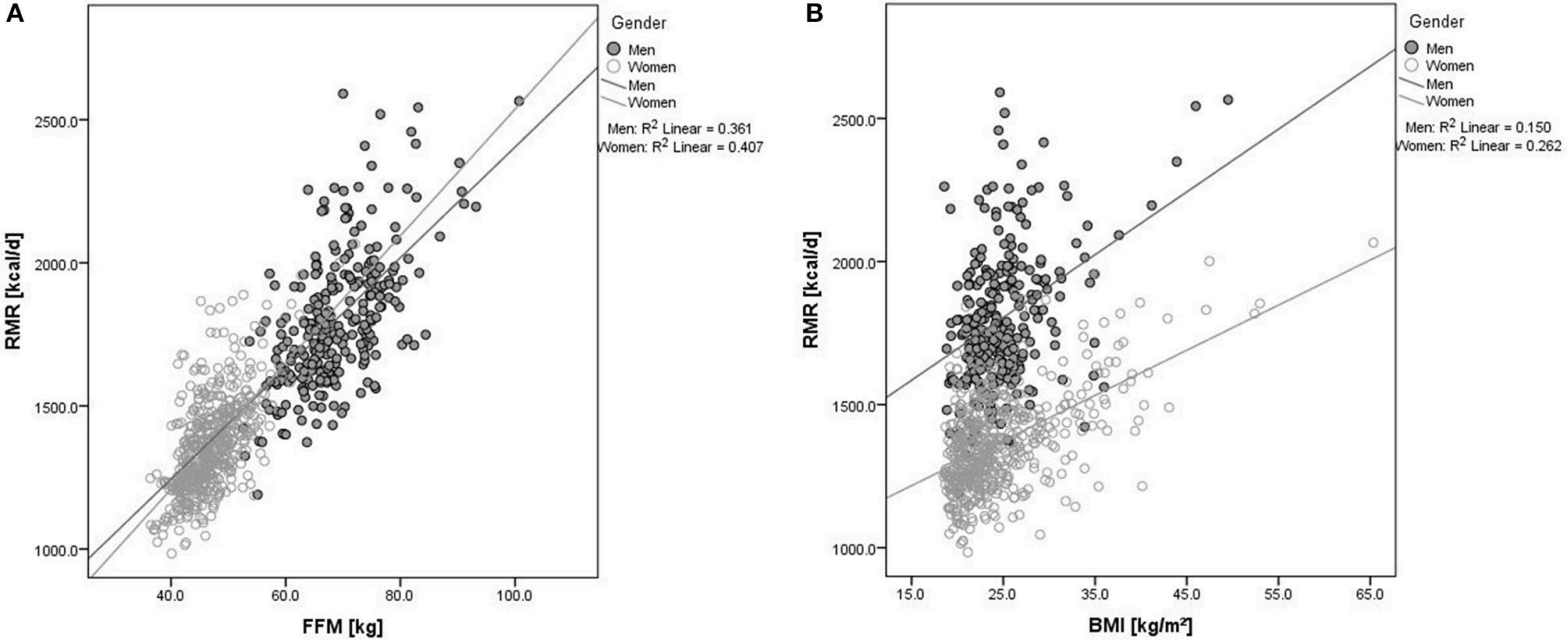
Association between RMR and **(A)** FFM or **(B)** BMI according to sex. Black dots represent data for males and open circles represent data for females. RMR, resting metabolic rate; FFM, fat-free mass; BMI, body mass index; R^2^, proportion of variance supposed by regression.

Significantly positive associations were observed between RMR and FFM (ß = 22.3 ± 0.8 kcal/d, *p* ≤ 0.001) after adjustment for age, sex, and study site and between RMR and BMI (ß = 8.8 ± 1.2 kcal/d, *p* ≤ 0.001) after adjustment for age, sex, FFM, and study site. Figure 1 shows sex-specific results. There was a significant association between RMR and FFM in both, males (ß = 20.7 ± 1.2 kcal/d, *p* ≤ 0.001) and females (ß = 24.1 ± 1.0 kcal/d, *p* ≤ 0.001) (Figure 1A). As shown in Figure 1B, similar results were observed for the association between RMR and BMI (males, ß = 9.9 ± 2.7 kcal/d, *p* ≤ 0.001; females, ß = 7.9 ± 1.3 kcal/d, *p* ≤ 0.001). A significantly positive association was observed between RMR and insulin levels (ß = 6.4 ± 1.2 kcal/d, *p* ≤ 0.001), but not between RMR and plasma-equivalent glucose levels (ß = 0.5 ± 0.5 kcal/d, *p* = 0.315).

### Mediator analysis

**Figure 2:**
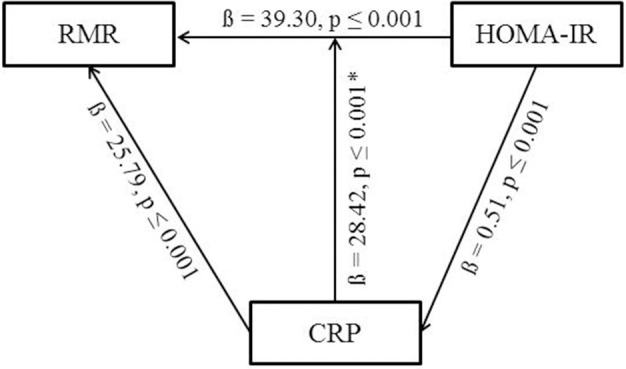
Overview of the mediator analysis. Results are adjusted for FFM, sex, age, study site. *Significant *p*-values do not change after including CRP into the model. RMR, resting metabolic rate; CRP, C-reactive protein; HOMA-IR, homeostasis model assessment for insulin resistance.

Results of the mediator analysis showed evidence for a significant association between RMR and HOMA-IR (ß = 39.3 ± 7.3, *p* ≤ 0.001) and between CRP and HOMA-IR (ß = 0.5 ± 0.1, *p* ≤ 0.001), with RMR as the dependent variable (Figure 2). Furthermore, a significant evidence was found for an association between RMR and CRP (ß = 25.8 ± 4.1, *p* ≤ 0.001) describing an additional result. The mediator analysis did not support the hypothesis of a mediating effect of CRP on the association between RMR and HOMA-IR in both unadjusted and adjusted analyses since HOMA-IR remained highly significant (adjusted: ß = 28.4 ± 7.5, *p* ≤ 0.001) after inclusion of CRP (Table 3). Further adjustment for smoking and physical activity did not change the results (Table 3).

**Table 3:** Mediator analysis for the unadjusted and adjusted models.

**Model**	**Dependent variable**	**Independent variable**	**Regression coefficient ± SE**	**95% CI**	***P*-value**
**MEDIATOR ANALYSIS (UNADJUSTED)**					
**1**	RMR	HOMA-IR_log_	83.88 ± 15.57	53.31; 114.45	≤0.001
**2**	CRP_log_	HOMA-IR_log_	0.57 ± 0.06	0.45; 0.69	≤0.001
**3**	RMR	HOMA-IR_log_	91.97 ± 16.43	59.72; 124.23	≤0.001
		CRP_log_	−14.23 ± 9.29	−32.46; 4.00	0.126
**MEDIATOR ANALYSIS (ADJUSTED FOR FFM, AGE, SEX, STUDY SITE)**					
**1**	RMR	HOMA-IR_log_	39.30 ± 7.29	24.98; 53.61	≤0.001
**2**	CRP_log_	HOMA-IR_log_	0.51 ± 0.06	0.39; 0.63	≤0.001
**3**	RMR	HOMA-IR_log_	28.42 ± 7.50	13.70; 43.14	≤0.001
		CRP_log_	21.18 ± 4.22	12.91; 29.46	≤0.001
**MEDIATOR ANALYSIS (ADJUSTED FOR FFM, AGE, SEX, STUDY SITE, SMOKING, PHYSICAL ACTIVITY)**					
**1**	RMR	HOMA-IR_log_	40.96 ± 7.51	26.22; 55.69	≤0.001
**2**	CRP_log_	HOMA-IR_log_	0.47 ± 0.06	0.35; 0.59	≤0.001
**3**	RMR	HOMA-IR_log_	30.79 ± 7.66	15.76; 45.82	≤0.001
		CRP_log_	21.70 ± 4.28	13.30; 30.10	≤0.001

*Beta estimates ± SE are shown; RMR, resting metabolic rate; HOMA-IR, homeostasis model assessment for insulin resistance; CRP, C-reactive protein; FFM, fat-free mass; BMI, body mass index; log, logarithmically transformed*.

Subanalysis according to BMI categories (normal-weight, BMI 18.5–24.9 kg/m^2^; overweight, BMI 25.0–29.9 kg/m^2^; obesity, BMI 30.0–40.0 kg/m^2^) did not show evidence for a mediator role of CRP (Table 4). Repeating the mediator analysis separately for persons with normal-weight (BMI < 25.0 kg/m^2^) and a merged group of participants with a BMI of equal to or >25.0 kg/m^2^ revealed that the association between RMR and HOMA-IR remained significant (ß = 50.5 ± 11.6, *p* ≤ 0.001) only in the group of persons with overweight or obesity (Table 4).

**Table 4:** Mediator analysis according to BMI groups adjusted for FFM, age, sex, study site.

**Model**	**Dependent variable**	**Independent variable**	**Regression coefficient ± SE**	**95% CI**	***P*-value**
**MEDIATOR ANALYSIS, BMI 18.5-24.9 kg/m^2^ (*****n*** = **511)**					
**1**	RMR	HOMA-IR_log_	18.90 ± 10.04	−0.82; 38.62	0.060
**2**	CRP_log_	HOMA-IR_log_	0.14 ± 0.08	−0.02; 0.03	0.084
**3**	RMR	HOMA-IR_log_	16.69 ± 9.99	−2.95; 36.32	0.096
		CRP_log_	15.76 ± 5.46	5.03; 26.48	0.004
**MEDIATOR ANALYSIS, BMI 25.0-29.9 kg/m^2^ (*****n*** = **182)**					
**1**	RMR	HOMA-IR_log_	39.11 ± 15.22	9.08; 69.14	0.011
**2**	CRP_log_	HOMA-IR_log_	0.48 ± 0.12	0.24; 0.72	≤0.001
**3**	RMR	HOMA-IR_log_	31.29 ± 15.81	0.08; 62.49	0.049
		CRP_log_	16.32 ± 9.54	−2.50; 35.13	0.089
**MEDIATOR ANALYSIS, BMI** ≥ **30.0 kg/m^2^ (*****n*** = **88)**					
**1**	RMR	HOMA-IR_log_	39.70 ± 19.73	1.04; 78.36	0.044
**2**	CRP_log_	HOMA-IR_log_	0.41 ± 0.15	0.10; 0.71	0.009
**3**	RMR	HOMA-IR_log_	30.91 ± 20.09	−9.07; 70.88	0.128
		CRP_log_	21.61 ± 13.97	−6.18; 49.40	0.126
**MEDIATOR ANALYSIS, BMI** ≥ **25.0 kg/m^2^ (*****n*** = **270)**					
**1**	RMR	HOMA-IR_log_	50.46 ± 11.60	27.62; 73.29	≤0.001
**2**	CRP_log_	HOMA-IR_log_	0.63 ± 0.10	0.45; 0.82	≤0.001
**3**	RMR	HOMA-IR_log_	37.08 ± 12.37	12.71; 61.44	0.003
		CRP_log_	21.19 ± 7.45	6.52; 35.86	0.005

*Beta estimates ± SE are shown; RMR, resting metabolic rate; HOMA-IR, homeostasis model assessment for insulin resistance; CRP, C-reactive protein; FFM, fat-free mass; BMI, body mass index; n, number; log, logarithmically transformed*.

No significant evidence for a mediator role of CRP was observed if participants with hypertension (>140/90 mmHg), with dyslipidaemia, with drug intake such as statins and aspirin, or with IFG levels were excluded (data not shown). A separate analysis including only participants with IFG (*n* = 85) showed no evidence for CRP as a mediator of the HOMA-IR–RMR association (Table 5). Results of interaction analyses did not show evidence for an interaction of BMI and HOMA-IR or BMI and CRP on RMR. Results were not significant with further analysis investigating the interaction between sex and HOMA-IR or sex and CRP on RMR (data not shown).

**Table 5:** Mediator analysis in a subgroup of participants with IFG adjusted for FFM, age, sex, study site.

**Model**	**Dependent variable**	**Independent variable**	**Regression coefficient ± SE**	**95% CI**	***P*-value**
**MEDIATOR ANALYSIS, (ADJUSTED FOR FFM, AGE, SEX, STUDY SITE)**					
**1**	RMR	HOMA-IR_log_	51.61 ± 21.82	8.19; 95.03	0.020
**2**	CRP_log_	HOMA-IR_log_	0.46 ± 0.19	0.09; 0.83	0.016
**3**	RMR	HOMA-IR_log_	42.19 ± 22.42	−2.45; 86.84	0.064
		CRP_log_	20.68 ± 13.12	−5.44; 46.79	0.119

*Beta estimates ± SE are shown; IFG, impaired fasting (plasma-equivalent) glucose; RMR, resting metabolic rate; HOMA-IR, homeostasis model assessment for insulin resistance; CRP, C-reactive protein; FFM, fat-free mass; BMI, body mass index; log, logarithmically transformed*.

## Discussion

The current analysis of a large cohort of adults with varying BMI confirmed that RMR was positively associated with FFM and BMI. Moreover, a positive association between RMR and HOMA-IR as a measure of IS, as well as CRP as a marker of the inflammatory status was observed. However, the mediator analysis suggested that CRP was not a mediator contributing to the association between RMR and HOMA-IR.

The positive association between RMR and HOMA-IR, which was reported in previous studies (8, 9, 25–28), was replicated in the current analysis even after adjustment for age, sex, and study site. The large sample size of the current analysis covering a broad range of BMI extends this observation. However, there is currently no convincing explanation for the association between RMR and HOMA-IR. Nevertheless, several proposed explanations include elevated protein metabolism, activated substrate cycle, increased gluconeogenesis and activation of the sympathetic nervous system, all potentially caused by hyperinsulinemia (8, 25, 29). In a cross-sectional study of 149 families, genetic predisposition, and obesity-related cardio-metabolic risk factors were reported to be associated with RMR variability (9). Furthermore, a positive association between fasting plasma glucose as well as impaired glucose tolerance and RMR was described (12, 30–32). The current analysis revealed a significant association between RMR and fasting insulin but provided no significant evidence for a positive association between RMR and fasting plasma-equivalent glucose in the adjusted model. Sub-analyses considering IFG levels of participants did not change the results and confirm that CRP is no mediator within the HOMA-IR–RMR association.

Another aspect is immunological function which requires up to 15% of daily energy expenditure (33). In clinical trials, CRP, an acute-phase plasma protein synthesized by liver, was shown to be an established systemic biomarker reflecting inflammation (5, 34). Most studies investigating the potential association between RMR and CRP were performed in severely ill patients, such as those suffering from chronic kidney disease (13), sepsis (35), or pancreatic cancer (36). An association between RMR and inflammation measured by CRP was shown in a cohort of healthy normal-weight and overweight Caucasians (28). This finding is in line with the significantly positive association between RMR and CRP found in the current analysis after adjustment for relevant confounders.

Obesity, impaired IS or T2DM are characterized by low-grade inflammation frequently assessed by CRP as well as interleukin 6 (IL-6) or tumor necrosis factor alpha (TNFα) (5, 6, 37). Regarding this, the current analysis provides support for the role of inflammation within the IS pathways based on the significant associations between HOMA-IR and CRP. This finding was previously described in a two-year weight loss study (38). Here, McLaughlin et al. observed that CRP levels of obese insulin-sensitive women were significantly lower than in obese, insulin-resistant women.

Nevertheless, the underlying causes of increased RMR in subjects with impaired IS or overt diabetes as well as the impact of an activated immune system are still debated, and the mechanisms are not fully understood. The present analysis showed significant associations between RMR, CRP and HOMA-IR. Therefore, it is of added value to investigate whether CRP mediates the association between RMR and IS, represented by HOMA-IR. However, the results of the present analysis did not reveal significant evidence for CRP as a mediator within the association between RMR and HOMA-IR. One possible explanation for this outcome might be related to the current sample consisting mainly of young and healthy adults, in contrast to other studies investigating the effect of CRP on RMR in cohorts with participants suffering from severe diseases (13, 14, 35, 36). Other studies argued that a CRP level above 1 mg/dl, which was associated with RMR, was also correlated with acute infections or severe chronic inflammation (39, 40). In the present study, participants with severe or acute diseases were excluded and, therefore, only a small percentage of the participants (2.4%) exhibited an elevated CRP level (>1.0 mg/dl). This small group (*n* = 19) represented a significantly higher mean BMI of 32.9 kg/m^2^ compared to the group with a lower CRP level (*p* ≤ 0.001). Therefore, BMI might itself play a crucial role in diabetes and the inflammatory status. Lending support to this possibility, subanalysis within the Chennai Urban Rural Epidemiology Study (CURES) found significant correlations between CRP, FM, and HOMA-IR (41). In addition, a cross-sectional study of an Israeli population found that subjects with obesity had markedly higher CRP levels than normal-weight persons (40). This finding is in line with a previous study by Yudkin et al. who reported that CRP levels were positively correlated with BMI in persons without diabetes (42). Furthermore, increased RMR is hypothesized to protect from further weight gain (43). From a biological point of view, IL-6 which is expressed in adipose tissue in addition to many other sites stimulates liver CRP production and leads to higher circulating CRP levels. Furthermore, fat cells produce and secrete more pro-inflammatory and less anti-inflammatory cytokines, particularly from enlarged adipocytes, which might represent the link to disturbances in glucose-insulin homeostasis and dysregulation in energy metabolism (4, 6, 37, 42). Nonetheless, in the current study, 23.3% of the participants were overweight and 11.3% were obese. Subanalyses of the data showed that the association between RMR and HOMA-IR remained significant within the merged group of participants with overweight or obesity compared to normal-weight persons. This finding suggests a possible interaction between BMI and HOMA-IR on RMR. However, interaction analyses failed to show significant evidence for BMI–HOMA-IR or BMI–CRP interaction on RMR. Furthermore, the results of the subanalysis showed no evidence for CRP as a mediator within the RMR–HOMA-IR association after BMI stratification.

## Strengths and limitations

This is the first analysis investigating the potential role of CRP as a mediator within the association between RMR and HOMA-IR in a large Caucasian population. For the mediator analysis the frequently used methodology of Baron and Kenny (24) was applied. This stepwise analysis allows to explore the underlying mechanisms of the association between RMR and IS. The analysis was based on a large cohort of well phenotyped, healthy subjects, and a quite homogenous dataset due to standard operating procedures and exclusion criteria. Several subanalyses, which confirmed results of the mediator analysis, were carried out. Therefore, confounding factors like smoking, physical activity, or IFG levels can be excluded. Nevertheless, it has to be mentioned, that the results are not representative for the general population.

RMR was measured by indirect calorimetry which is considered as the gold standard for measuring energy expenditure (44). In the present analysis, inflammation was assessed by circulating concentrations of CRP, which has a low specificity but is widely accepted as a robust marker of the inflammatory status. However, CRP levels were measured once, whereas a two-point measurement of CRP levels is usually recommended to avoid within-person variability among patients with stable metabolism (39). Replication studies as well as studies considering further inflammatory markers such as IL-6 or TNFα are necessary.

Moreover, HOMA-IR was used as an indirect parameter of IS. Participants with reported overt diabetes and fasting plasma-equivalent glucose levels above 125 mg/dl were excluded from the dataset. Further established methods to determine the diabetes status such as an oral glucose tolerance test or hemoglobin A1c measurement were not used. As CRP and HOMA-IR were not normally distributed, values were log-transformed for calculation, and the possible interference of the log values cannot be ruled out. In addition, even if the criteria for each stage of a mediator analysis were given it is still unclear whether the non-significant association between RMR and fasting plasma-equivalent glucose values has an impact on the results.

## Conclusion

The current analysis provided significant evidence for an association between RMR and IS in a large cohort of healthy adults. Conversely, there was no significant evidence for a mediator role for circulating CRP within the association between RMR and HOMA-IR. The findings were not conclusive due to the known low specificity of CRP and likely due to the healthy and young study population. Further studies are needed to replicate these findings and investigate the largely unexplained association between glucose metabolism and energy expenditure.

## Author contributions

TD and TS performed the measurements. TD was in charge of the statistical analysis of the data. TD and CH processed the experimental data, contributed to the idea, and design of the research, performed the analysis, drafted the manuscript, and designed the figures and tables. Therefore TD and CH share the first authorship. LS gave statistical advice, aided in interpreting the results and worked on the manuscript as a native speaker. JP and TS contributed to the design and implementation of the research and to the writing of the manuscript. HH designed and supervised the research project. All authors discussed the results, commented on the manuscript, and approved the final version to be submitted.

### Conflict of interest statement

The authors declare that the research was conducted in the absence of any commercial or financial relationships that could be construed as a potential conflict of interest.

## Abbreviations

T2DM: type 2 diabetes mellitus
IS: insulin sensitivity
RMR: resting metabolic rate
(hs-)CRP: (high sensitive) C-reactive protein
BMI: body mass index
IFG: impaired fasting (plasma-equivalent) glucose
HOMA-IR: homeostasis model assessment for insulin resistance
VO_2_: volume oxygen
VCO_2_: volume carbon dioxide
SD: standard deviation
FFM: fat-free mass
IL-6: interleukin-6
TNFα: tumor necrosis factor alpha.
